# Patients’ experiences with a community fruit and vegetable box program prescribed by their health provider

**DOI:** 10.1186/s12889-023-15685-w

**Published:** 2023-05-11

**Authors:** Jennifer K. Johnson, Evelyn Vingilis, Amanda L. Terry

**Affiliations:** 1 Centre for Studies in Family Medicine, Department of Family Medicine, Schulich School of Medicine and Dentistry, Western University, 1151 Richmond St, London, ON N6A-5C1 Canada; 2 Population and Community Health Unit, Department of Family Medicine, Department of Epidemiology and Biostatistics, Schulich School of Medicine and Dentistry, Western University, 1151 Richmond St, London, ON N6A-5C1 Canada; 3 Centre for Studies in Family Medicine, Department of Family Medicine, Department of Epidemiology and Biostatistics, Schulich Interfaculty Program in Public Health, Schulich School of Medicine and Dentistry, Western University, 1151 Richmond St, London, ON N6A-5C1 Canada

**Keywords:** Food insecurity, Fruit and vegetable prescriptions, Focus groups, Community dietician, Healthcare provider

## Abstract

**Background:**

Food insecurity is “the state of being without reliable access to a sufficient quantity of affordable, nutritious food”. Observational studies have associated food insecurity with many negative health effects including the development and exacerbations of chronic diseases, higher health care use and increased mortality. Health care providers prescribing food is a growing area of interest and research, however it is not known how patients feel about receiving fruit and vegetable prescriptions (FVRx) from their health provider versus other means of food provision. This pilot study was conducted to explore the experiences and opinions of Canadian adults with food insecurity who were recipients of a FVRx box program prescribed by their health provider.

**Methods:**

Potential participants were recruited to 3 focus groups using flyers included in their monthly food box. Questions were kept open to encourage participation of all group members. The focus groups were audiotaped, transcribed verbatim, and analyzed by the research team using descriptive qualitative research methodology.

**Results:**

Participants described shame and frustration trying to obtain enough food through local food banks. In comparison, they perceived their team dietitian, family physician or addictions physician as directly helping them with their health by prescribing food. The boxed fruit and vegetables were prepared in many ways and often shared to reduce waste and to reduce the food insecurity of extended family members. Positive effects of the FVRx on physical and mental health were reported. Participants believed that follow up with their health provider helped support them and their behavioural changes towards better nutrition. Limitations of the program included lack of choice, non-flexible pick-up times and the program being limited to 6 months. Being able to choose their own fruit and vegetables, instead of receiving a set box, was suggested by most to help meal planning and to increase autonomy.

**Conclusions:**

Health providers prescribing FVRx boxes to adult patients with food insecurity was positively received in this study. Evaluation of similar programs in other regions in Canada and internationally, and comparison of food prescriptions to basic income guarantee programs is recommended.

## Background

Food insecurity is defined as “the state of being without reliable access to a sufficient quantity of affordable, nutritious food” [1]. However, theories have evolved over time, and it has been proposed by the United Nations’ High Level Panel of Experts on Food, Security and Nutrition that food security frameworks not only include the 4 dimensions: availability, access, utilization, and stability, but also include the 2 dimensions: sustainability and agency [2].

Observational studies have associated food insecurity with greater health care use and costs, including increased neonatal intensive care admissions, increased mental health service utilization and reduced prescription adherence [3, 4]. Food insecurity has also been associated with gestational diabetes, asthma in children, mobility limitations in older adults, reduced exclusive breast feeding, postpartum mental health disorders and increased mortality [5–8].

Globally, food insecurity has been rising, with marked increases since the COVID-19 pandemic. In 2021, 29.3% of the global population was estimated to have moderate or severe food insecurity [9].

Prescribing food to people through health care systems is a growing area of interest and research [10]. Food prescription programs have been used to target childhood obesity [10], prenatal health [11], hypertension [12], type 2 diabetes [13, 14], dietary behaviour in youth [15, 16] and food insecurity in depressed patients [17]. Fruit and vegetable prescription (FVRx) programs appear to be effective at engaging patients with health messages including the association between diet and health [18]. They also likely reduce food insecurity [19].

Primary care providers have expressed satisfaction prescribing food [18, 20], but how patients feel about receiving fruit and vegetable prescriptions (FVRx) from their health provider versus other means of food provision has not been well explored, particularly in Canada. A recent systematic review of food prescription programs in primary care found very few studies that explored the patient perspective. Furthermore, none of the 23 studies included in the review were conducted in Canada [19].

### Project context

The Karma Project has been providing local food to North Simcoe, Ontario since 2007 through food boxes and grocery vouchers funded by local government grants. The Project’s vision of integrating healthy food into the health care system started in 2017 with the idea of engaging local health care providers in prescribing fruit and vegetables to their patients who would benefit. In 2020, Karma ran a pilot feasibility FVRx program with the delivery of fruit and vegetable boxes and vouchers to the homes of 23 families recruited by the North Simcoe Family Health Team’s dietitians, one local family physician and one addiction medicine physician. The goal was to replicate the prescribing of food as medicine to counter challenges with food access, the cost of healthy food, food literacy and transportation barriers. Initial informal feedback was positive. A more in-depth understanding of patients’ experiences with the FVRx program was sought to support a continuous quality improvement process in 2021 with 50 families participating. The aim of this pilot study was to explore the experiences of participants recruited for the 2021 FVRx program.

## Methods

### Ethics approval

for this study was obtained from the Office of Human Research Ethics at Western University. (Project ID #118,458)

### Participants

Patients 18 years or older who were interested in the FVRx program were referred by their participating health provider (community family physician, addiction medicine physician, or health team dietitian) if they screened positive for food insecurity using the Hunger vital sign tool [21]. The Hunger Vital Sign is a 2-item questionnaire with convergent validity that is used to identify families and individuals with food insecurity. This tool asks how often the household ‘worried whether food would run out before we got money to buy more’ and how often ‘the food that we bought just didn’t last and we didn’t have money to get more’ [19]. An affirmative answer of ‘often true’ or ‘sometimes true’ to either question 1 or 2 has a sensitivity of > 97% and a specificity of > 74% for food insecurity in adults [22].

### Recruitment

The fifty families enrolled in the program were offered participation in the study through recruitment posters added to their monthly food box; twelve individuals who agreed to participate and met the inclusion criteria were enrolled in the study. The flyers included a brief explanation of the study and the contact email address and telephone number of the study Principal Investigator (PI). Interested participants who contacted the PI were sent the study letter of intent (LOI) and consent form. Individuals who were able, emailed the signed consent back to the PI before the focus group. Those participants who were not able to email back a signed consent, gave their consent on the phone (sent a text or called study cell phone), and then signed a consent form in person at the time of the focus group.

All participants received a $20 grocery gift card honorarium for participating in the study.

### Data collection

Experiences of recipients of the FVRx boxes were explored using focus groups. Focus groups are a useful exploratory research methodology for conducting an initial examination of a topic for which there is limited information [23–25]. An additional advantage of focus groups over individual interviews is that the interaction among group members can stimulate clarification of views [26, 27]. Focus groups were chosen as a method to give participants the opportunity to express and to exchange viewpoints on shared experiences (in this case, food distribution programs) that could not be obtained with individual interviews.

In keeping with qualitative methods, questions were purposely kept open and unstructured to encourage participation of all members. Using an interview guide, the focus group participants were asked to share their experiences getting, preparing and eating fruit and vegetables, for their thoughts about the food box program, and how the program affected them. They were also asked for their experience with other food programs, how the food box program could be improved, how they felt about their health provider suggesting the program and how their health provider could best help them improve their nutrition.

The focus groups were taped using two audio-recorders and transcribed verbatim. One of the authors served as the group facilitator (JJ). Field notes were made by this facilitator to capture non-verbal communication, and her observations during the focus groups. All focus groups were conducted outside with social distancing protocols in place because of the COVID-19 pandemic between July - October, 2021.

### Study design

Descriptive qualitative research methodology was chosen as the most suitable method for analyzing the data because this method emphasizes accurate, in-depth reporting of conversations as they happened [24, 25].

### Data analysis

The research team (JJ, EV, AT) initially coded the transcripts from each focus group independently with keywords and phrases for each element of text. These independent interpretations of the data were discussed and compared when the team came together after each focus group and modified collectively to form a single coding template that reflected the consensus of the groups’ insights and interpretations. This iterative process of applying the coding to new focus groups continued until saturation was reached and no new themes or concepts were identified as agreed upon by the team.

Trustworthiness and credibility were promoted by various means: the interview transcripts were transcribed verbatim and reviewed for accuracy; the team met regularly for debriefing meetings to discuss the field notes taken after each focus group; and reflexivity was practiced by reflecting on emerging themes, alternate interpretations and possible biases [28]. Regular meetings of the researchers allowed for discussion of individual team members’ perspectives and professional backgrounds on the study design, data interpretation and concluding findings.

Positionality and reflexivity are important to attend to when conducting qualitative research. They also help the reader understand the contextual relationship between the focus group facilitator (study investigator JJ) and the participants [29]. The facilitator is a female family physician who is interested in food insecurity and how primary care might reduce the impact of food insecurity on health. She had referred several patients from her family practice to the F&V program in the previous year (2020), but had not received feedback from these patients about the program.

The facilitator was introduced to the participants as a researcher, open to and interested in their views on food programs, but separate from the food box program and separate from her role as a family physician in the community. The facilitator of the focus groups did not prescribe F&V in 2021 and was not a family physician to any of the participants. These were intentional strategies to reduce the power differential. However, the potential vulnerability of the participants was recognized by the facilitator.

Participants were reassured that their responses were confidential and that the facilitator had no influence on the program or whether they were, or would continue to be, food box recipients.

## Results

The demographics of the participants recruited to the study are shown in Table 1.

**Table 1:** Participant Demographics (n = 12; 3 focus groups of 4 participants each)

Age Range	Gender		Referring Health Provider	
30–69	Female	10	Family physician	3
(Mean age 49)	Male	2	Dietician	6
	Other	0	Addictions Physician	3

As can be seen in Table 1, the majority of participants were female and participants were referred equally between physicians and dietitians. This is also true of the sum total of food box recipients involved in the program. 75% were women, and 50% were referred by either their addictions physician or family physician. Therefore, the study sample was reflective of the FVRx program.

Five main themes emerged from analysis titled *“Living With Food Insecurity”, “Shame, Humiliation and Frustration With Food Charities”, “Benefits of a FVRx Program”, “Feeling Supported” and “What if We Could Purchase Our Own Food?”.*

### Living with food insecurity

The focus group participants described not being able to afford fruit and vegetables on their limited income: *“I have a lot of health issues…we go from pay to pay so when it comes down to payday, by the time we pay everything, I really can’t afford to buy a lot of fresh fruit and vegetables.” (P8).* They also reported constant worry of not having enough money to buy any food, nutritious or not: *“… it’s your very strict budget…it never makes it to the end of the month. You’re always scrambling at the end, going, ‘What am I going to make to eat now?’” (P3)*.

Some participants gave instances of going without food or sacrificing eating healthy food so that other family members could have it:

I’ll eat stuff that I wouldn’t give to my kids because I want my kids to eat good food, right? So, whatever’s left over during the week I’ll eat it. Moldy bread, moldy buns, pick it off…sometimes if the meat is so-so, I’ll eat it but I won’t feed it to my kids. (P1)

They also described the challenge of having to constantly make trade-offs between buying food versus other necessities:

It’s challenging to make your grocery list every week…you’ve got to sacrifice here or there, right? Like what do I lose? TV, internet? You can’t stop paying your rent. You need rent and food. (P1)

The rising costs of food added to the stress the focus groups reported experiencing: *“I’ve noticed with the prices now of fruits and vegetables have just gone drastically through the roof. Everything has gone right through the roof. It’s like how does anybody expect us to live?” (P10)*.

The findings illustrate the significant hardships of not being able to regularly obtain healthy food.

### Shame, humiliation and frustration with food charities

The second theme describes the participants’ experiences with food banks and other food box programs. The focus group participants described experiences of powerlessness associated with having to depend on food programs which sometimes meant having to accept poor quality food: *“when you’re just given second-rate food and make do…How does that make you feel*?” *(P1)*. Despite needing it, another participant described not being able to eat any of the donated food: “*I stopped that after two or three times because the food in it was just too rotten.” (P3)*.

Several participants described sometimes receiving negative treatment at food banks: *“I went there at church and the lady yelled at me and said, ‘Are you even supposed to be here?’” (P1).* Another participant remarked: *“They make you feel like you’re a criminal, like you’re stealing it or something.” (P2).*

One participant described feeling guilty about going to a food bank: *“I don’t know. I just… I kind of felt just guilty, because I kept thinking I was taking from people that could – you know, that were in worse shape than I was.” (P10).* Another participant reported feeling embarrassed at being scrutinized: *“you’re basically standing in this huge line-up with a bunch of other people, you’re basically on display” (P4).*

The contributions of the study participants suggest that obtaining food from charities does not completely relieve food insecurity and may add to their stress.

### Benefits of a FVRx program

The third theme describes the focus groups’ experiences with the FVRx food program.

Compared to programs that supply canned food, the focus group participants described enjoying the fresh fruit and vegetables: *“The flavour, simple texture, pleasure of texture of eating something that’s supposed to be eaten the way it should be, not all mush, right?” (P1).*

The participants described valuing time together preparing and cooking the fresh fruits and vegetables as a family: “*I think it’s good too, just for the family aspect, healthy, doing things together. Like getting the box together is exciting for everybody to go through, right?” (P6).*

The focus groups reported improvement in chronic health problems experienced by family members:

I noticed for my daughter…she was having issues with her diabetic readings… And now that she’s getting more fruits and vegetables, they’ve come down again so she’s doing a lot better there… (P4)

Regarding her overweight son, another participant remarked:

He’s overweight. So, I think it’s a really good thing because it’s all healthy foods, right? So, it’s kind of an exciting thing that he’s getting this [the FVRx food box] and then we can kind of work on what we’re going to eat, how we’re going to cook it and things like that. (P6)

Some participants described less food insecurity with the FVRx food box: *“It gave me a sense of security actually…on that date I was going to have lots of fruit and vegetables in my fridge and it made me feel like a million bucks, and I could plan some healthy meals.” (P9).*

### Feeling supported

The fourth theme reflects how the focus groups responded to being referred to this FVRx program by their health provider. Most focus group participants responded positively to their health provider referring them to the FVRx program: *“I was so appreciative my doctor called and asked me if I’d be interested, I was like ‘Oh!’ and I started to cry. Thank you so much.” (P9)*.

Only one participant felt embarrassed at being identified as having food insecurity by their health provider, *“I was embarrassed at first and I didn’t want to do it because I don’t like handouts right… I just don’t like asking for help or people thinking I need help.” (P8).*

Generally, the participants stated that they appreciated having their health provider involved. They believed that it was appropriate for their health provider to promote and support good nutrition to improve their health in this way: *“I think that doctors should be prescribing more holistic and natural things as opposed to just giving out a pill all the time.” (P7).*

One participant expressed the wish that there was more follow through from their health provider after they were referred to the FVRx program: *“They just have so many patients… but it would be nice for them to check up on people.” (P6)*.

### What if we could purchase our own food?

The final theme reflects the focus group participants’ suggestions and ideas for lessening their food insecurity. The idea of going to a grocery store to buy food using a discount card instead of receiving food boxes was raised by one participant: *“… instead of all the money going into the box programs, all this stuff, the grocery stores are there. You just go and buy your groceries or your vegetables and your meat products or whatever… you can get on [a discount] card”(P1).* The other participants reflected on this suggestion, and contributed to the discussion on how to improve food distribution in the future:

*“Yeah, then you can pick what you want. because there’s a lot of things in the box that I don’t use.” (P3)*.

*“Well yeah, like rutabaga or, and”(P1)*.

*“Yeah I hate that stuff”(P3)*.

*“Mm-hmm, overabundance of lettuce”(P4)*.

*“You’ve got a huge head of lettuce but what are you going to eat with it, right?”(P1)*.

*“Exactly, and how fast can you use it up before it goes bad?”(P3)*.

One participant disagreed: *“I’m going to be honest with you, I think if I had the card, I’d probably buy something that wasn’t needed…like a block of cheese or something” (P12).*

While most of the participants expressed a preference for choosing their own food, some saw benefits of a delivered food box.

## Discussion

Food insecurity is increasing and becoming more severe in Canada [30] and worldwide [31] since the COVID-19 pandemic. This qualitative study explores the perspectives of food insecure adults who were recipients of a Canadian pilot community FVRx box program.

There are two unique findings in this study. The first is the importance of privacy in food distribution programs. Participants described feeling shame, humiliation or frustration relying on food charities such as food banks. Most commented that using these programs meant they had to accept food that was often poor quality. Moreover, food bank staff did not always consider their need for privacy regarding their food insecurity. Yet, confidentiality about a patient’s food insecurity was reported as important to both patients and health care providers in a study by Nederveld et al. [31].

The second key finding of this study is the need for recipients to have more control over food choices. This supports the viewpoint that agency should be considered when developing programs that address food insecurity. Participants’ suggestions for program improvement focussed on their desire to choose fruit and vegetables in contrast to being given a set food box. In addition, regarding food banks, most commented that using these programs meant they had to accept food that was often poor quality. Participants believed there would be less waste if they could redeem FVRx prescriptions at grocery stores where they could pick preferred, higher quality produce in amounts needed for family meal planning. As described by Little et al. [19], this expressed wish for more autonomy reflects a tension in the patient-healthcare provider relationship: by striving to improve patient health equity through food prescriptions, health providers risk reinforcing the paternalistic tradition of dictating how care, in this case food, is provided to patients. Empowering patients to participate in improving and sustaining their health and to form a more equitable patient-provider relationship has been emphasized in primary care [32] of late. Perhaps being prescribed a guaranteed income or at the very least, food vouchers, would be more empowering for food insecure patients than receiving a food box. A basic income guarantee has been proposed as the most effective strategy to reduce food insecurity [33].

Participants described experiences of food insecurity that are universally stressful. For example, parents reported sacrificing eating nutritious food by giving much of what was available to their children. Other studies have observed similar efforts: of the 20% of food insecure homes in the US in 2020, approximately 50% of parents were able to shield their children from food insecurity [34].

Additionally, participants remarked that receiving fresh fruit and vegetables facilitated more opportunities to prepare healthier food with their children. This is an important finding because early adoption of healthy eating patterns is recognized to have lifelong impacts [35]. Furthermore, time cooking together in food insecure homes, may also improve the mental health of family members through the strengthening of important relationships [36]. This is relevant because a recent systematic review concluded that food insecurity is associated with increased risk of depression and stress [37].

Participants reported that the community FVRx program provided both physical and psychological benefits. Consistent with a recently published systematic review that concluded food prescriptions likely increase fruit and vegetable intake and reduce food insecurity [19], participants described improved nutrition with the food box program. They perceived positive impacts on their family’s chronic health problems such as diabetes, obesity and mental illness. Research has similarly found that food prescriptions may improve diabetic control [13, 14], hypertension [12] food insecurity with depression [17], weight [10] and food insecurity in general [19].

The participants were generally pleased that their health provider inquired about their interest in being referred to the FVRx program. They perceived an improved relationship with their health provider, particularly with those providers who followed up with them after referral. Previous studies have shown that the most effective programs at increasing intake of fruits and vegetables included follow up nutritional education [12, 18, 38].

In the absence of a national or provincial basic income guarantee program, health care providers may be in a position to identify, and refer to food programs, those patients with food insecurity. This means that health care providers in Canada would need to collect data on all their patients regarding their social determinants of health. While these efforts are likely to be challenging in clinical practice, given many health care providers are currently overburdened, it would allow screening for patient eligibility when programs such as FVRx are made available to primary care providers or dietitians. As well, clinical encounters where social determinants of health are discussed may deepen the patient-healthcare provider relationship [39].

### Limitations

This study is limited by participants being recruited from only one FVRx program in one region of Ontario, Canada. The sample was also predominantly female possibly because women are more comfortable participating in focus groups than men, more women in this region of Ontario have food insecurity, or women are more likely to find and participate in community food programs. Previous research suggests that because women are typically more responsible for shopping and meal planning, there is higher utilization of food programs by women [40]. As well, women are more likely to experience food insecurity. Currently one third of female led single parent families in Canada have food insecurity [41]. Finally, some FVRx recipients may have avoided participating in this study because of fear of contracting the COVID-19 virus despite public health measures being followed. (Virtual focus groups were not considered because many participants did not have video capabilities.)

### Implications for research and practice

There is growing interest in primary care addressing social determinants of health such as poverty and food insecurity experienced by their patients [42]. There is also interest in produce prescriptions between the health care sector, community organizations and farmers markets [16]. Advocates for produce prescribing propose that individual interactions with health care could provide opportunities for nutrition education and food interventions that lead to reductions in healthcare costs and use[43]. Currently, food programs are not coordinated with the healthcare system in Ontario.

## Conclusions

To date, there have been few qualitative studies of patients’ experiences receiving fruit and vegetable prescriptions in Canada. This pilot study provides insights into experiences of food insecurity, food charities, a food box FVRx prescription program, health provider involvement and participants’ ideas for improving access to healthy food from the perspective of patients with food insecurity. Findings from this study support the importance of privacy and agency when designing food programs. Further evaluation of FVRx programs, particularly in comparison with FVRx vouchers for grocery stores or farmers markets or with basic income guarantee programs is needed to determine which method is associated with greater satisfaction, less food insecurity and better health parameters.

## Data Availability

The qualitative datasets generated and/or analysed during the current study are not publicly available due to the data containing information that could compromise research participant privacy/consent but are available from the corresponding author on reasonable request.

## List of abbreviations

FVRx: Fruit and vegetable prescription
